# Moderate Multidimensional Poverty Index: Paving the Way Out of Poverty

**DOI:** 10.1007/s11205-023-03134-5

**Published:** 2023-05-27

**Authors:** Sabina Alkire, Fanni Kövesdi, Elina Scheja, Frank Vollmer

**Affiliations:** 1 Oxford Poverty and Human Development Initiative (OPHI), Department of International Development, University of Oxford, Oxford, UK; 2 Swedish International Development Cooperation Agency (Sida), Sundbyberg, Sweden

**Keywords:** Multidimensional poverty, Sustainable development goals, Poverty measurement, Intrahousehold inequality, Middle-income countries, Capability approach

## Abstract

Eradicating poverty in all its forms, everywhere, requires indicators that measure sustainable pathways out of poverty, and not only the absence of acute poverty. This paper introduces a trial Moderate Multidimensional Poverty Index (MMPI) that reflects moderate rather than acute levels of multidimensional poverty. The MMPI adjusts nine of the 10 indicators of the global Multidimensional Poverty Index (global MPI) to reflect moderate poverty and create a meaningful superset of the acutely poor population. Although data-constrained, the trial MMPI outlines a methodology and potential indicators for a measure that would: (i) be meaningful and comparable across populations at higher levels of development; (ii) align with higher standards defined in Agenda 2030; and (iii) provide insight into aspects of intrahousehold deprivation. The MMPI is illustrated empirically using nationally representative household surveys from Bangladesh, Guatemala, Iraq, Serbia, Tanzania and Thailand. The results confirm the added value of having three nested measures of destitution, acute, and moderate multidimensional poverty. The MMPI also complements monetary measures with informative differences in poverty levels observed. The results demonstrate that a Moderate MPI is a desirable global poverty index, which is likely to illuminate thus far hidden aspects in of multidimensional poverty, such as intrahousehold deprivations in education. Challenges remain regarding data availability, and further study across additional countries is required before the MMPI structure can be finalised.

## Introduction

The 2030 Agenda for Sustainable Development and the Sustainable Development Goals (SDGs) set a high standard for human development with the pledge ‘to end poverty and hunger, in all their forms and dimensions, and to ensure that all human beings can fulfil their potential in dignity and equality and in a healthy environment’ (United Nations General Assembly, 2015). This vision implies a life free from abject poverty, but also a reality where all are empowered to lead a life they value, make their own choices, and reach their full potential. The first and overarching goal of this agenda is to end poverty in all its forms, everywhere.

In many countries the headcount ratio using the global Multidimensional Poverty Index (global MPI)—which focuses on acute poverty—is in low single digits as a result of prudent policy making and improvement of basic capabilities (Alkire et al., 2020a, 2020b). While progress towards these basic capabilities is laudable, the 2019 *Human Development Report* called attention to widening inequalities in reaching *enhanced* capabilities that are required to ensure a life free from poverty and social exclusion as it is understood in most societies. Reaching the minimum floor of attainment, reflected in the global MPI and other acute measures, is insufficient in a world where enhanced capabilities are required for obtaining productive employment and being part of society at large. Although pre-pandemic, the world was about to reach the target of universal primary education, large inequalities persist in access to secondary education that provides functional skills for today’s labour market. Similarly, many people now have mobile phones, but lack access to smart phones, broadband or other forms of internet access that provide access to information and services.

A new measure of poverty is thus needed to capture the higher levels of ambition that align with the Agenda 2030 goals. This paper proposes a new Moderate Multidimensional Poverty Index (MMPI) that builds upon existing measures of acute poverty and creates a set of indicators that capture, insofar as data permit, *enhanced* capabilities for human development. The MMPI has three important strengths compared to previous efforts of measuring poverty: (i) it introduces a *global* measure that could be meaningfully compared across countries in all income categories, thus setting a realistic and universal norm for global debates on poverty; (ii) it aligns the human development indicators with the higher level of ambition outlined in the SDGs; and (iii) it includes an indicator to capture intrahousehold gender inequalities.

The trial MMPI is applied to six countries to illustrate the proposed methodology for creating a moderate MPI that is linked to the global MPI (where everyone who is poor according to the global MPI is still identified as poor), and uses the same datasets. Although a range of indicator combinations were empirically trialled, further investigation of the data for the full set of available countries is required in order to finalize the indicator structure of a proposed MMPI.

The paper is organized as follows: Sect. 2 describes the background and motivation for the new measure; Sect. 3 introduces the Alkire-Foster method, the global MPI and related indices and sub-indices, the main features of the proposed moderate MPI, and the data; Sect. 4 presents the empirical findings using the new index in six pilot countries; Sect. 5 draws conclusions from the empirical results and discusses further elaborations of the work in a broader policy framework.

## Background and Motivation

### Monetary Poverty

Many current poverty measures used to track progress towards SDG Goal 1 fall short of its ambition to end poverty in all its forms. Instead, poverty is most often measured in monetary terms only, using the international extreme poverty line established by the World Bank (2022). The $2.15 a day^1^ line is an average of national poverty lines in the poorest countries and covers the minimum set of basic needs. However, even in low-income countries, this line does not correspond to the level needed for sustainable poverty reduction ‘in all its forms, everywhere’, and in most middle-income countries, national poverty lines are higher, reflecting the higher level of income required to meet basic needs in these societies.

The World Bank applies two additional international poverty lines to better capture absolute poverty with respect to the relatively higher living standards in middle-income countries. The $3.65 a day (2017 PPP) poverty line reflects poverty in a typical lower middle-income country, while the $6.85 a day (2017 PPP) line reflects upper middle-income country standards. The World Bank also introduced a societal poverty line (SPL) that considers the median level of consumption in a country,^2^ and moves up as incomes rise (World Bank, 2022).

According to the 2019 global estimates, while 8.4% of the world’s population lived on less than $2.15/day, this was nearly a quarter (23.5%) for the higher poverty line of $3.65/day, and close to half (46.7%) of the world’s population for the $6.85/day measure.^3^

### Multidimensional Poverty

Ending poverty in all its forms, the aim of SDG 1, entails viewing poverty not solely in relation to income and consumption, but as relating to multiple capabilities such as health, education, and living standards. However, while the SDG indicator framework includes both the global $2.15/day and national monetary poverty lines to monitor progress towards ending monetary poverty, only national definitions of multidimensional poverty are included, preventing cross-country comparisons.^4^ However, a global measure of multidimensional poverty exists, and provides vital comparable information for 6.1 billion people in 2022.

The global MPI, developed by the Oxford Poverty and Human Development Initiative (OPHI) and the United Nations Development Programme (UNDP), has been updated regularly since 2010. It was developed to measure ‘acute’ multidimensional poverty globally and reflect aspirations in the Millennium Development Goals (MDGs). The global MPI measures poverty in terms of health, education and living standards using 10 indicators, and complements existing international monetary poverty measures. It was revised in 2018, at the start of the Third United Nations Decade for the Eradication of Poverty (2018–2027), to better align the indicators with the SDGs and Agenda 2030, insofar as data permit (Alkire & Jahan, 2018; Alkire et al., 2018).

The global MPI remains relevant for many countries where the incidence of acute poverty remains high. But in 2022, there are 41 countries where the incidence is below 5%, and it is under 1% in 21 countries (Alkire et al. 2022). Thus, the global MPI cannot fully capture the higher aspirations of governments and citizens in settings where acute deprivations have already been minimized or eradicated. As noted by Sen (1983), *relative* deprivation in terms of commodities, incomes and resources relates to *absolute* deprivation in terms of capabilities. In other words, while deprivation in all measured indicators is defined in absolute terms, they translate into differences in terms of capabilities depending on the context. For example, obesity might not be an indicator of poverty in all countries, and in some contexts, a lack of internet access may not imply poverty. In other contexts, the same deprivation can effectively exclude people from crucial interactions, opportunities and basic services, and thereby constitutes an important dimension of poverty.

A new measure of ‘moderate’ multidimensional poverty is therefore needed to complement the global MPI, and to capture deprivations in countries with low levels of acute MPI, as well as urban regions and low-poverty subnational provinces. Such a measure, together with the global MPI, can help to ensure that ‘all human beings can fulfil their potential in dignity and equality and in a healthy environment’ (United Nations General Assembly, 2015) and live a life free from all forms of poverty, as envisioned in Agenda 2030.

### Moderate Multidimensional Poverty

Previous exercises have explored measures of moderate poverty. The precursor to the global MPI, the Human Poverty Index (HPI) (Anand & Sen, 1997), had two forms—HPI-I and HPI-II—precisely to provide measures that were relevant across the entire distribution of countries. Alkire et al. (2019a) trialled a Middle-Income Countries MPI with new indicators (including employment) in six Latin American countries but found comparability challenging even for those countries. Similarly, the Multidimensional Poverty Measure (MPM) introduced by the World Bank in 2018 added human security as a new and exciting dimension, but data were only available for six countries. Some regional indices have also been developed to cater for similarities across countries. MPIs have been estimated from the EU-SILC databases (see Alkire & Apablaza 2017; Alkire et al. 2021), and regional MPIs were published for Latin America (Santos & Villatoro, 2016) and the Arab region (UN ESCWA, 2017). These indices include higher levels of achievement for the global MPI indicators, and/or add additional indicators and dimensions reflecting the realities of poverty in the region. Naturally, their structure and comparability is limited by data constraints, and does not replace the need for an internationally comparable measure of moderate poverty.

The new trial index presented in this paper, the MMPI, builds on the basic capabilities of the global MPI and introduces enhanced capabilities anchored in the SDGs that are needed for sustainable poverty reduction. For instance, the *years of schooling* indicator increases the level of ambition from basic education for one member of the household, to lower secondary education for at least one male and one female member of the household of working age, to proxy functional skills needed for employment. The MMPI builds on the universal standards for poverty reduction agreed in Agenda 2030, allowing better monitoring of progress at levels beyond acute deprivation. By raising the deprivation cutoffs for the indicators, the MMPI aims to parallel the higher poverty lines for monetary poverty and provide a meaningful gradient for sustainable development. Context matters greatly for how different deprivations play out across countries, and the MMPI is expected to be relevant and realistic (although sometimes aspirational) for all countries, although likely most meaningful for countries and subnational provinces where acute poverty is already low and possibly no longer reflects a valid level of ambition for development.

## Methodology and Data

### Methodology

The proposed MMPI takes as its starting point the revised global MPI, using the same methodology and structure. Both use the Alkire-Foster method, a flexible counting-based approach that allows poverty to be measured using dimensions, indicators and weights that reflect context and normative decisions (Alkire & Foster, 2011). After counting the weighted deprivations for each person or household, a *cross-dimensional poverty threshold* is applied to determine the minimum deprivation score at which a person or a household is considered multidimensionally poor. Poor people’s deprivation profiles are then summarized in a single index (the MPI) characterizing the overall level of deprivation across the population By counting the simultaneous weighted deprivations across indicators that each poor person or household experiences, the MPI can show directly how different conditions reinforce each other, creating poverty traps or deprivation bundles that are difficult to break without a clear understanding of the interlinkages.

Poverty is reported by the single headline measure of MPI, or the *adjusted headcount ratio* (MPI = *H* × *A*), composed of the *incidence* or headcount ratio (H), the proportion of people identified as multidimensionally poor, and the *intensity* or average deprivation share (A), that is the average share of (weighted) deprivations faced by multidimensionally poor people. By incorporating the intensity of poverty, the MPI goes beyond the simple headcount ratio used by monetary measures to consider gains among the poorest of the poor who have not yet exited poverty. Many governments aim to eradicate poverty by reducing the number of people living in poverty; however, they must also ensure that progress is being made to improve conditions for all—for instance, by reducing the average deprivation scores of the poorest people, thus bringing them a step closer to leaving poverty behind.

The MPI can be disaggregated by regions and population subgroups to better understand the inequalities in poverty, and broken down by indicator to look at the composition of poverty across the population and different subgroups. The *censored headcount ratio* presents the percentage of people who are poor and deprived in each component indicator, and the weighted sum of the censored headcount ratios are also equal to the MPI. The *percentage contribution* of each indicator to the MPI reflects the censored headcount ratio *and* the weight assigned to each indicator, showing the relative value of each component to the MPI. Finally, the *uncensored headcount ratio* aggregates the deprivations of poor and non-poor people to show the proportion of the entire population deprived in each indicator.

### Global Multidimensional Poverty Index

The best-known application of the Alkire-Foster method is the global MPI. Grounded in Amartya Sen’s capability approach, the measure focuses on human capabilities, understood as people’s real opportunities to do and be what they value and have reason to value (Alkire & Santos, 2014). It includes 10 indicators (see Table 1) grouped into three dimensions (health, education, and living standards) that reflect the Human Development Index. A nested weighting structure is applied, with all dimensions given equal weights (1/3), and all indicators within a dimension weighted equally. A person is considered to be multidimensionally poor if they are deprived in at least a third of the weighted indicators (33.33% or more) (Alkire et al., 2022).

**Table 1:** Dimensions, indicators, and deprivation cutoffs for the global MPI. *Source*: Alkire et al. (2020a, 2020b)

Dimension	Indicator	A household is deprived if:	Relative weight
Health	Nutrition	Any person under 70 years of age, for whom there is nutritional information, is malnourished	1/6
	Child mortality	A child under 18 years of age has died in the family in the five-year period preceding the survey	1/6
Education	Years of schooling	No household member aged 10 years or older has completed six years of schooling	1/6
	School attendance	Any school-aged child is not attending school up to the age at which he/she would complete grade 8	1/6
Living standards	Cooking fuel	A household cooks with dung, agricultural crops, shrubs, wood, charcoal or coal	1/18
	Sanitation	A household’s sanitation facility is not improved (according to SDG guidelines) or it is improved but shared with other households	1/18
	Drinking water	A household does not have access to improved drinking water (according to SDG guidelines) or safe drinking water is at least a 30-min walk from home (as a round trip)	1/18
	Electricity	A household does not have electricity	1/18
	Housing	A household has inadequate housing: the floor is made of natural materials or the roof or walls are made of rudimentary materials	1/18
	Assets	A household does not own more than one asset (radio, TV, telephone, computer, animal cart, bicycle, motorbike, refrigerator) and does not own a car or truck	1/18

### Related Measures: Severe Poverty and Destitution

The dual-cutoff approach of the Alkire-Foster method can be used to distinguish two differently defined subsets of people living in poverty. One set is identified by changing the poverty cutoff (intensity approach), and the other by changing the vector of deprivation cutoffs for given indicators (depth approach) (Alkire & Seth, 2016).

First, the level of ambition can be changed by altering the cross-dimensional poverty cutoff, that defines the minimum percentage of weighted deprivations required for a person to be identified as multidimensionally poor. This can range from being deprived in one indicator (union approach) to being deprived in all indicators (intersection approach). The global MPI poverty cutoff is k = 33.3%, and two additional cutoffs are also reported, for ‘vulnerability’ (k = 20%) and ‘severe’ multidimensional poverty (k = 50%). The latter identifies a *subset*, the poorest of the MPI-poor. Everyone who is severely poor is also MPI-poor by design (Alkire et al., 2020a, 2020b).

Another way of identifying the poorest of all poor people is to change the deprivation cutoffs of the indicators. The ‘destitution’ measure (Alkire et al., 2014) identifies people as destitute by using more extreme criteria in 7 of the 10 global MPI indicators, for instance severe undernutrition instead of undernutrition, open defecation instead of a lack of basic sanitation, or one year of schooling instead of less than 6 years (Alkire et al., 2020a, 2020b). As the adjusted vector is used with the same weights and poverty cutoff, everyone who is destitute is also MPI-poor by definition, forming an alternative *subset* of MPI-poor people.

While OPHI developed the destitution measure in 2014, a corresponding moderate measure could not be designed due to serious data limitations at that time. With improvements in many of the surveys used for the global MPI, in 2020 the decision was made to reassess the construction of a global MMPI. Like the destitution measure, the proposed MMPI uses the global MPI dimensions, indicators and weighting structure, but instead of looking at a subset of poor people, it adopts the depth approach to capture a *superset* of moderately poor people. By changing the vector of deprivation cutoffs for 9 of the 10 indicators, the MMPI identifies *additional* households as moderately poor, who lack the enhanced capabilities required for a life free from poverty as defined in Agenda 2030.

### Moderate Multidimensional Poverty Index

Retaining the global MPI structure enables comparisons and facilitates communication, as the main structure is widely known in international policy fora. Drawbacks of keeping to the established dimensions of the global MPI include the limited choice of indicators, and the exclusion of some aspects of poverty that are decisive for people living in countries with higher income or development levels. But as was established earlier, while most national multidimensional poverty indices in middle-income countries include employment as an important dimension (see Angulo et al., 2016), employment could not be included for a comparable measure (Alkire et al. 2019a). Like the global MPI, the MMPI is geared towards defining a set of universal, enhanced capabilities required to enable people to move out of poverty in any given country or regional context. The choice of the modified indicators is based on the proposal for comparable policy-relevant indicators by Atkinson (2019). However, limited availability of data has restricted the set of possible indicators to a subset of desirable indicators, and means that the MMPI presented here is a trial measure.

The proposed indicators (Table 2) build on the global MPI definitions with deprivation cutoffs raised (e.g. school attendance) or new conditions added (e.g. electricity) to reflect higher levels of ambition. Any person or household identified as deprived by the global MPI indicator is automatically also deprived by the MMPI indicator. The modified deprivation cutoffs, when integrated with the same poverty cutoff of 33.33%, identify *additional* people as poor, thus creating a new *superset* of moderately poor people.

**Table 2:** Dimensions, indicators, and deprivation cutoffs for the MMPI (in bold) based on the global MPI. Source: Alkire et al. (2022), modified by authors to reflect MMPI

Dimension	Indicator	A household is deprived if:	Relative weight
Health	Nutrition	Any person under 70 years of age, for whom there is nutritional information, is malnourished **or obese**	1/6
	Child mortality	A child under 18 years of age has died in the family in the five-year period preceding the survey or not all eligible household members are covered by health insurance^*^	1/6
Education	Years of schooling	**No man and no woman** aged 10 years or older in the household has completed **nine years** of schooling	1/6
	School attendance	Any school-aged child is not attending school up to the age at which he/she would complete **grade 10**	1/6
Living standards	Cooking fuel	A household cooks with dung, agricultural crops, shrubs, wood, charcoal or coal	1/18
	Sanitation	A household does not have **flush toilet** that is not shared with any other household	1/18
	Drinking water	A household does not have access to **safe piped water on the premises**	1/18
	Electricity	A household does not have electricity or does **not have access to the internet or a smartphone**	1/18
	Housing	A household has inadequate housing: the **floor or roof or walls are made of natural or rudimentary materials** or there are **more than three people per sleeping room**	1/18
	Assets	A household does not own more than **two assets** (radio, TV, telephone, computer, animal cart, bicycle, motorbike, refrigerator, **washing machine, bank account**) and does not own a car or truck	1/18

^*^If data were available for two eligible household members (a man and a woman), only one member needed to report health insurance for the entire household to be considered covered

#### Education

A key dimension in all existing multidimensional poverty measures, education is widely considered a basic, but enabling, human right (UNESCO, 2016) due to its close links to other dimensions of wellbeing, such as employment, health and social participation. It is clearly linked to public policy and education deprivations are central to poverty. People with more years of schooling are found to be more productive and have higher incomes (World Bank, 2018, pp. 38–9) as well as better supporting nutritional and educational needs of household members. Yet wide inequalities persist in access to and completion of secondary education, and poorer households are still significantly disadvantaged (UNESCO, 2015).

Recent studies have also highlighted intrahousehold differences in educational achievements and participation, with women and girls having lower mean years of education than their male counterparts (Alkire et al., 2019a, 2019b). Agenda 2030 targets require countries to remove gender disparities in education, increase the skill levels of the adult population, and ensure that all children complete ‘12 years of free, publicly funded, inclusive, equitable primary and secondary education—of which at least nine years are compulsory’ (United Nations, n.d.-a).

In the global MPI, educational achievement is measured using two indictors: one focusing on educational attainment of the household members (*years of schooling*) and one relating to *school attendance* among children (if any) of the household. As shown in Table 1, none of the global MPI education indicators require household members to be enrolled or have achieved any secondary education (beyond grade 8), falling short of the goal of a minimum of nine years of free compulsory public education across the world (UNESCO, 2016).

The MMPI therefore includes revised cutoffs for both indicators that capture the level of ambition in the SDGs, and intrahousehold inequalities in educational achievements. In the MMPI, a household is considered deprived if it does not have at least *one man and one woman*^5^ of working age (16–60 years old) who had completed nine years of schooling, or if the household’s children do not attend school up to grade 10, which in many countries marks the completion of lower secondary education. In households with no working-age adults, this is lowered to six years of schooling for up to two eligible members, recognizing that elderly household members may not have had the same possibilities for schooling as younger generations. This new specification allows us to identify gaps in education up to the level where household members can be expected to have functional and marketable skills, without constraining educational achievement over time, based on historic access to education.

The new MMPI specification also sheds light on gendered disparities in educational deprivation by requiring the same achievement by at least one male and one female household member. It is the first such attempt to account for intrahousehold levels of achievements in an internationally comparable poverty index. The revised indicator recognizes that inequalities in education inhibit progress in poverty reduction and eradication, and celebrates progress in narrowing this gap. Importantly, the new indicator design will cause the uncensored headcount ratio in years of schooling to be higher in societies with greater gender disparities in education, underscoring the importance of achieving gender equality in the fight against poverty.

An important caveat from the global MPI also applies to the educational dimension of the MMPI. While years of schooling provides an important indication of educational achievement, it does not reflect the quality of schooling and learning outcomes. This is an important part of development outcomes for which comparable data are not yet available in the household surveys used to calculate the global MPI or the MMPI, so they are unable to capture this aspect of learning. However, quality of schooling should be carefully considered when drawing policy recommendations.

#### Health

Prior to the COVID-19 pandemic, there had been great progress in improving health outcomes globally, for example in rapidly declining cases of child and maternal mortality, albeit at varying speeds in different parts of the world (UNDP, 2019, p. 39). Challenges remain in widespread access to essential health services that could ease the burden of communicable disease, and reduce non-communicable health issues (e.g. diabetes and cardiovascular disease) that are gaining ground, especially in upper middle- and high-income countries (WHO, 2020). In the global MPI, deprivation in health is measured by undernutrition (underweight and, for children, stunting) and child mortality, both of which capture acute forms of health deprivations. While they remain an issue, many countries have successfully reduced or eliminated these acute conditions (Alkire et al., 2020b).

The MMPI adds two additional criteria to the existing health indicators of the global MPI:

(1) *Health insurance,* to capture access to healthcare in line with SDG Target 3.8 on universal health coverage; and (2) *Obesity* to proxy the increasing burden of non-communicable diseases. Not having access to healthcare can deprive people of the opportunity to remain healthy and employable, leading to new poverty traps and deprivations in other dimensions of poverty (Banerjee & Duflo, 2011). Promoting universal health coverage links to many other target areas, such as reducing poverty (SDG 1), universal educational access and better-quality education (SDG 4), advancing gender equality (SDG 5), inclusive growth and productive employment (SDG 8) and inclusive societies (SDG 16) (WHO & World Bank, 2017).

Although the SDG standard defines malnutrition as both over- and underweight, the MMPI includes a stricter definition, focusing on malnourishment and obesity instead of overweight, to proxy the increasing burden of non-communicable diseases, and the rapid increase in obesity as a major health problem in developing and middle-income countries. According to WHO, ‘worldwide obesity has nearly tripled since 1975’, and today, ‘most of the world’s population live in countries where overweight and obesity kills more people than underweight’ (WHO, 2021)—a condition exacerbated by the coronavirus pandemic. In the MMPI, obesity is measured using the WHO definition: for adults, as body mass index (BMI) greater than or equal to 30; for young people (aged 5–19) as BMI-for-age greater than two standard deviations above the WHO Growth Reference median; and for children under 5 as weight-for-height greater than three standard deviations above WHO Child Growth Standards median (WHO, 2021). Broadening the definition of the nutrition indicators aligns these with SDG 2 that strives to end hunger, achieve food security, improve nutrition and promote sustainable agriculture.

Notable data limitations exist in the expanded health-related indicators due to sample coverage and variation in the modules administered in each country. For obesity, data limitations prevent the MMPI from capturing the full scale of the problem in countries with Multiple Indicators Cluster Surveys (MICS) that only record children’s nutritional status. Since obesity is mostly a problem later in life, this can lead to underreporting of the nutrition deprivation in countries with MICS. Similarly, the coverage of health insurance is limited in MICS, with no data in Bangladesh, Serbia and Thailand, and only partial information in Iraq (women only). This lowers the accuracy of the revised indicator, with ramifications for the interpretation and cross-country comparability of the results.

It is also important to note that health insurance—as used in the MMPI—is an imperfect proxy for our primary object of interest (the capability to live a healthy life), and that the absence of health insurance can imply very different deprivations across contexts. In countries where universal healthcare is provided by the government, no insurance is needed to obtain access. In other countries, insuring one member of the household may provide access to all household members even if this is not explicitly stated in the data. Finally, even with valid health insurance, health care of decent quality is not always available. We retain health insurance in this trial MMPI despite the current data limitations for normative reasons, due to the upcoming wave of household surveys in which data on health insurance will be captured, possibly in a more reliable manner.

Alternative health indicators that also feature in the global agenda, such as smoking and vaccination coverage, were considered in trial measures but none proved appropriate as a general indicator of population health and they were therefore excluded from the final specification.

#### Living Standards

Living standards are measured in the global MPI using six indicators (Table 1) and the MMPI increases the level of ambition for five of these (Table 2).

For sanitation and drinking water, the MMPI indicator requires a higher SDG standard of facilities. Thus, a household is considered deprived if it does not have a flush toilet that is solely for private use (i.e. not shared with other households). For drinking water, the MMPI requires the household to have piped drinking water that is on the premises. This brings the indicator close to SDG 6 on safely managed drinking water, defined as an ‘improved basic drinking water source which is located on premises, available when needed and free of faecal (and priority chemical) contamination’ (UN, n.d.-c), although data on measured water purity and disruption to water services is not available for inclusion in the MMPI.

For the housing indicator, the MMPI adds a new criterion to the definition of adequate housing. According to UN-HABITAT (2006, pp. 70–1), overcrowding is a hidden form of homelessness that affects mainly urban dwellers, and tends to be less prevalent as areas develop and gain increasing prosperity. Adding overcrowding to the MMPI aligns the indicator with the SDG definition of adequate housing, according to which ‘a dwelling unit provides sufficient living area for the household members if not more than three people share the same habitable room’ (UN, n.d.-b).

The MMPI maintains the rigorously tested assets schedule, defined in the revision of the 2018 global MPI (Vollmer & Alkire, 2022), as a minimum floor. It adds a washing machine (a higher-end item) and a bank account (as a proxy for access to finance) to the list of assets, as both items that can be associated with greater material wealth, particularly in middle-income countries. To accommodate increasing the assets from nine to 11 items, the minimum threshold for being non-deprived is raised to two items,^6^ with households expected to have more and better assets. It is important to note that, in some countries, mobile phones rather than bank accounts are used for financial transactions; elsewhere, bank accounts may be opened automatically (e.g. to receive social transfers) even if some account holders cannot properly use them or access to bank branches or internet is limited. Nevertheless, financial inclusion through a bank account or similar is frequently found to be an important pathway out of poverty (Shepherd et al., 2019) in many countries, and SDG 8 highlights the importance of financial access with its aim to ‘expand access to banking, insurance and financial services for all’.

For electricity, the global MPI defines a household as deprived if it does not have electricity. While lack of electricity is a sign of deprivation, finding pathways out of poverty requires *using* electricity for productive purposes to enhance one’s employability, resource base or income, and to access information. As data on electricity usage and interruptions are scarce, the MMPI cannot directly improve the indicator. To reflect the increased level of ambition and the importance of access to information and the role of communication technology in the fight against poverty, the MMPI considers a household to be deprived if it does not have electricity and has no access to the internet or a smartphone. The internet can provide access to public and market information, and offer means to protect fundamental freedoms, as reflected in SDG 9, that strives to ‘provide universal and affordable access to the Internet in least developed countries by 2020’. Moreover, the COVID-19 pandemic has refocused attention on the centrality of internet access to other parts of life such as remote working or virtual schooling. Including this indicator in the MMPI allows decision makers to effectively track the digital divide and ensure that nobody is left offline. However, note that data do not clarify whether the household has reliable internet coverage or sufficient data bundles on the cell phone.

The final living standards indicator in the global MPI is cooking fuel, providing a proxy for indoor air pollution and respiratory health by assessing reliance on unclean fuels for cooking and heating. Being non-deprived—not using any of the listed fuels—ensures cleaner indoor air and thus removes the source of deprivation. No grading of alternative cooking fuels is deemed necessary in this regard, and the MMPI retains the original deprivation threshold (Table 2).

### Data

The MMPI was calculated for six countries (Table 3) that vary in their level of economic development (according to the World Bank’s income classification) and cover six world regions. These illustrative results are not intended to provide a representative sample of all country contexts, but do show how the MMPI functions across a diversity of contexts.

**Table 3:** Typology of countries. *Source*: Authors’ compilation

Country	Type of survey	Year of survey	Income category^a^	Region
Bangladesh	MICS	2019	Lower middle-income	South Asia
Guatemala	DHS	2014/15	Upper middle-income	Latin America & the Caribbean
Iraq	MICS	2018	Upper middle-income	Middle East & North Africa
Serbia	MICS	2014	Upper middle-income	Europe & Central Asia
Tanzania	DHS	2015/16	Lower middle-income	Sub-Saharan Africa
Thailand	MICS	2015/16	Upper middle-income	East Asia & the Pacific

^a^ Based on the World Bank classification (1 July 2020 release); see World Bank (2021)

Like the global MPI, the MMPI relies on data from nationally representative household surveys: Multiple Indicator Cluster Surveys (MICS), and Demographic and Health Surveys (DHS). While both provide information on multiple aspects of poverty, the surveys have somewhat different objectives. In MICS, nutritional data are only collected for children under 5 years old, whereas DHS also collect nutritional data for men and women. There is also variation—both between DHS and MICS, and across countries—in the questionnaires and modules included, and the sample population for certain variables, such as health insurance or internet access. The empirical findings and cross-country comparisons should be analysed with these differences in mind. Additional details on data comparability are in the Appendix (Table 8).

## Results

### Revised Indicators

A first question is what impact the additional indicator criteria had on measured deprivations. It is important for policy makers to distinguish what is driving the results in these combined indicators in order to draw relevant conclusions for policy. This section provides some examples.

**Fig. 1:**
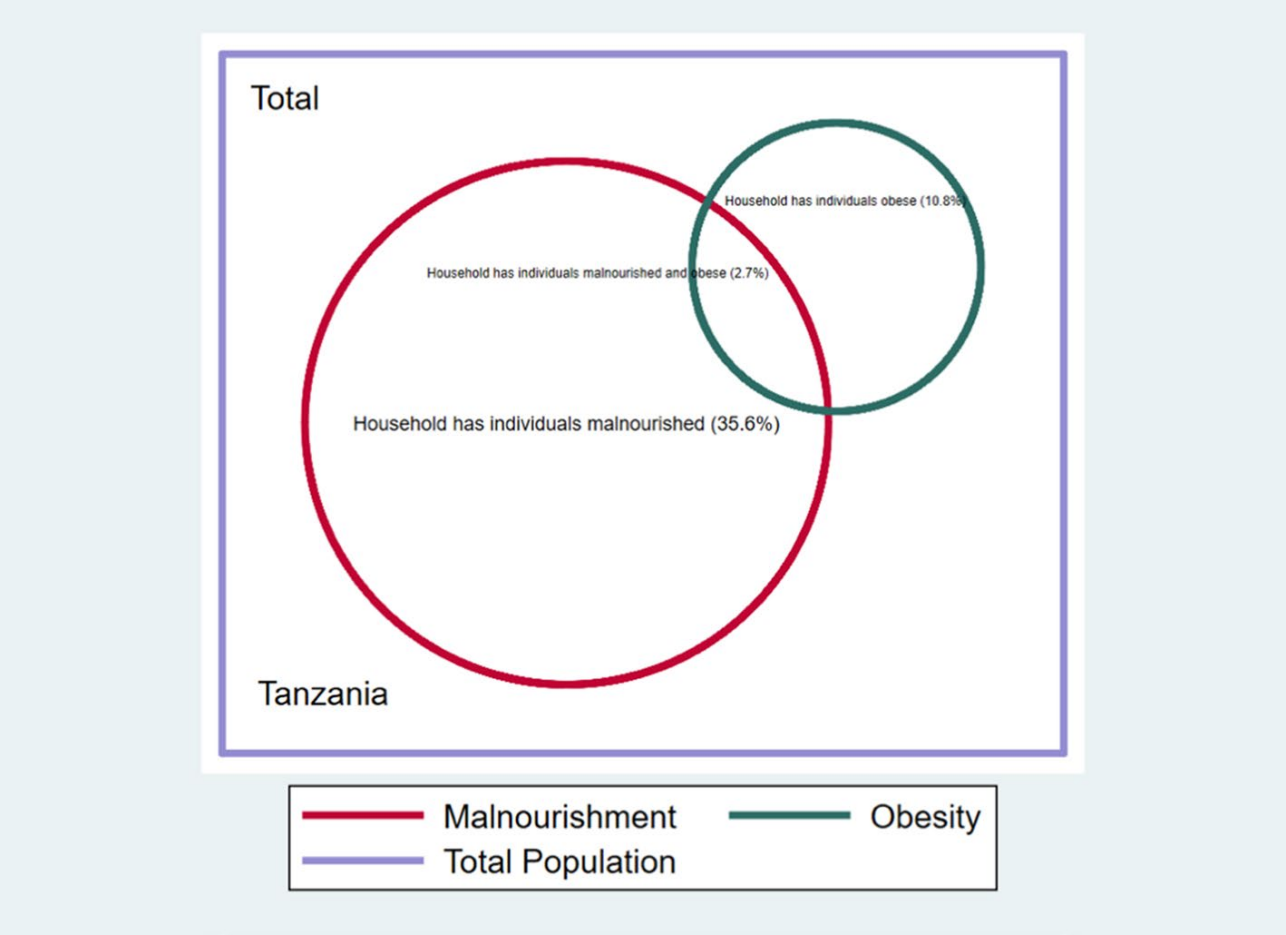
Undernourishment and obesity (Tanzania). *Source*: Authors’ calculations. Percentages refer to the eligible reference population with observable data only

For some indicators, the components are almost mutually exclusive—such as finding undernourishment and obesity in the same household. For example, Fig. 1 presents a Venn diagram of the percentage of people living in households that have only malnourished, only obese members, or both, in Tanzania. Only 2.7% of households for whom information on malnourishment and obesity were available have both types of deprivations within the same household. Although low, this should be read in the context of an increasing global trend of a double burden of malnourishment and obesity in the same households in many low- and middle-income countries (Branca et al., 2020).

**Fig. 2:**
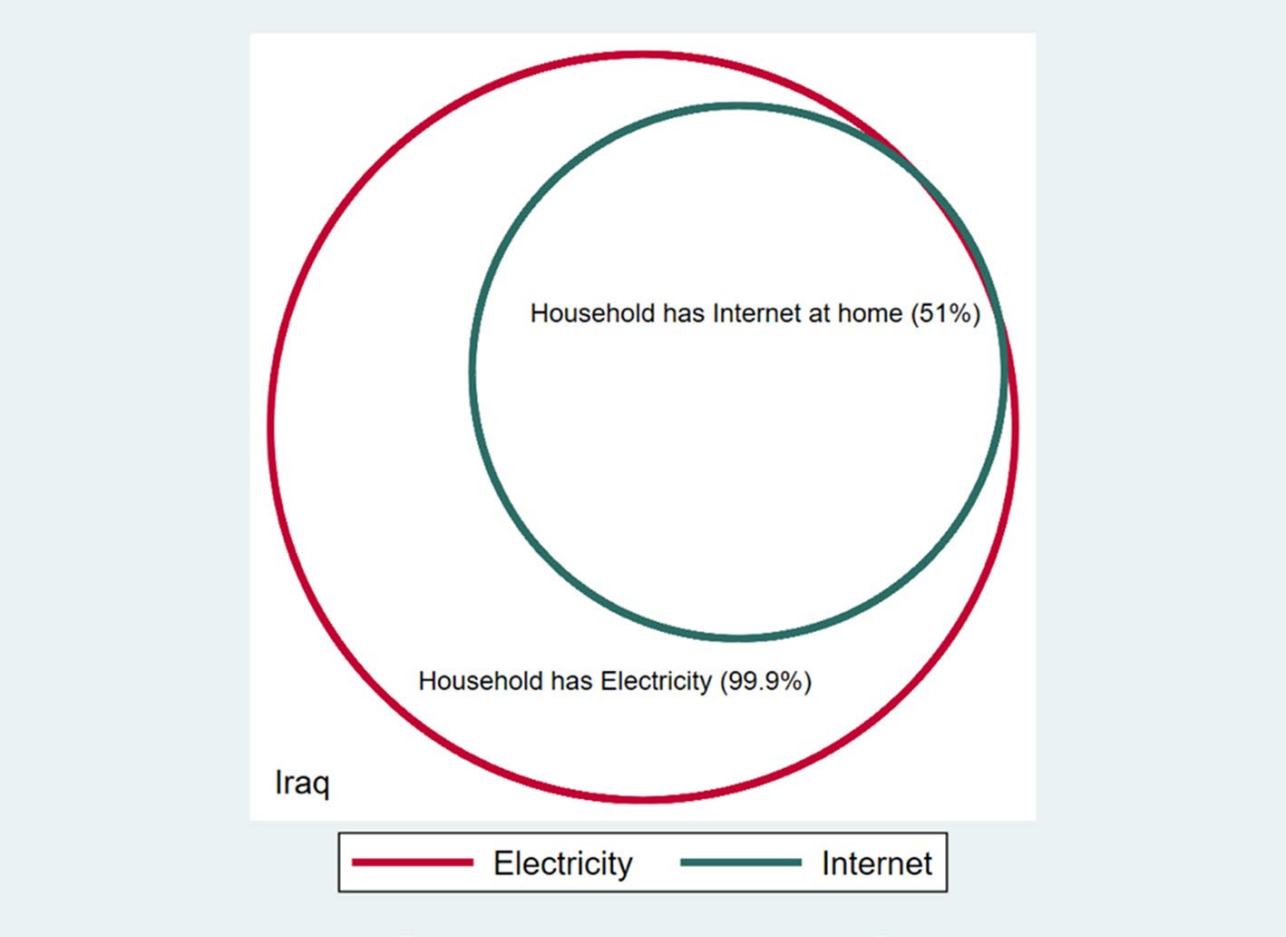
Electricity and internet (Iraq)**.** *Source*: Authors’ calculations. Percentages refer to the eligible reference population with observable data only

Other conditions can often be complementary, such having electricity and internet access, as electricity is a prerequisite for being able to use the internet in the household. This can be seen in the Venn diagrams for Iraq (Fig. 2), where everyone living in households with internet also have access to electricity.

**Fig. 3:**
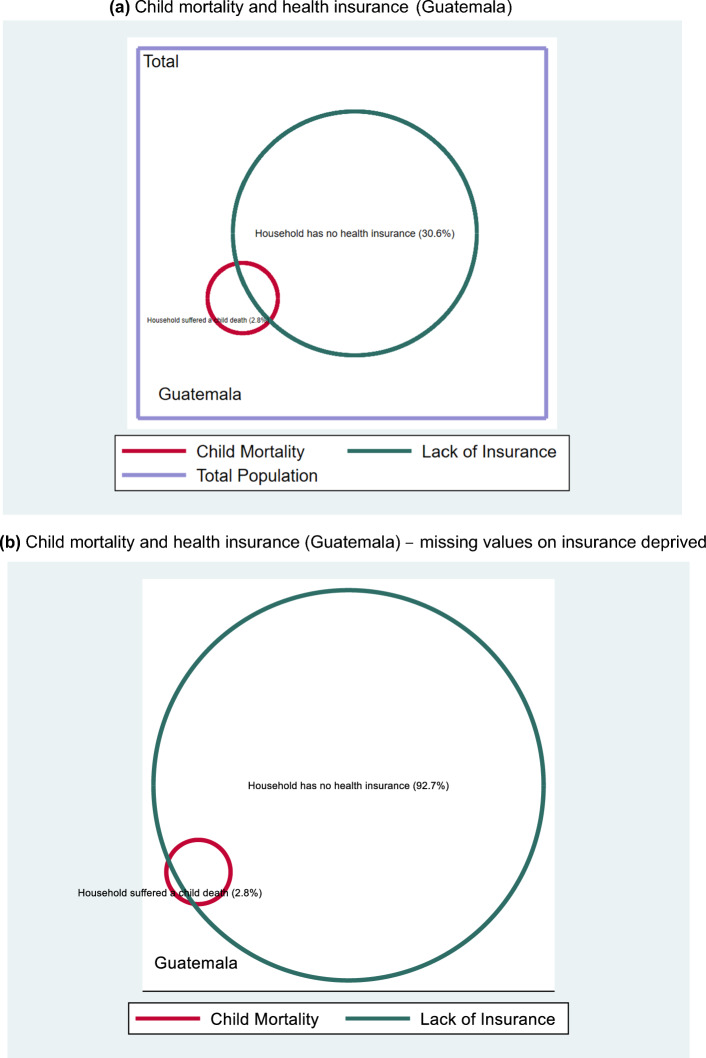
**a** Child mortality and health insurance (Guatemala)**.** *Source*: Authors’ calculations. Percentages refer to the eligible reference population with observable data only. **b** Child mortality and health insurance (Guatemala) – missing values on insurance deprived. *Source*: Authors’ calculations

In other indicators, the incidence of deprivation by the global MPI standard is so low that the added component to the indicator will dominate the MMPI results. This is the case for the combined indicator of child mortality and health insurance. In Guatemala (Fig. 3a), while only 2.8% of the population live in households that suffered a child death in the past five years, 30.6% of people live in households where no members have health insurance.^7^ This gap increases even further once the 63.6% missing values that are excluded from the Venn diagram in Fig. 3a but are included in Fig. 3b, are interpreted as deprivations (as is done in the MMPI, see Sect. 4.2). The notable difference in terms of Venn diagram interpretation is that once this assumption is implemented, the conditions on child mortality and insurance are rather complementary, similar to Fig. 2, meaning that almost all individuals living in households that suffered a child death in the past 5 years also lack health insurance.

Overall, driven by the addition of a new condition, the MMPI indicator is capturing a larger segment of the population as deprived, compared to the original global MPI indicator.

### Comparisons with the Global MPI

**Fig. 4:**
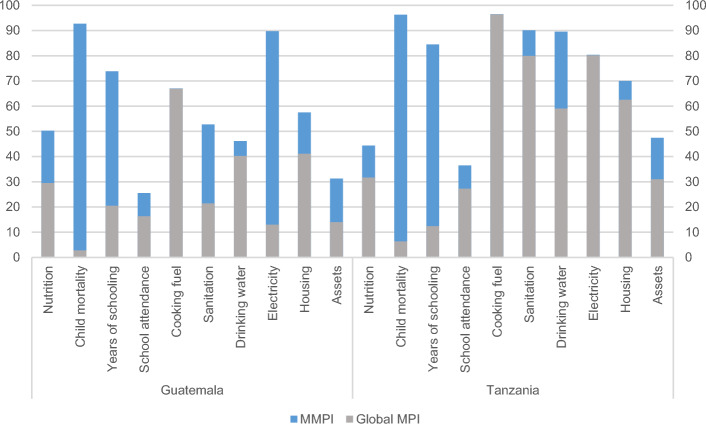
Uncensored headcount ratios for Guatemala and Tanzania (%). *Source*: Authors’ calculations. Global MPI estimates taken from Alkire et al. (2020a, 2020b)

This section analyses the changes across all nine indicators for which deprivation cutoffs were altered. Figure 4 depicts the changes in the proportion of people deprived in each indicator for each measure in Guatemala and Tanzania (see Table 9 in the Appendix for details for all countries). Overall, changing the indicator definitions led to substantial changes in the uncensored headcount ratios in most countries.

With the health dimension indicators, including obesity as part of the nutrition indicator caused an increase in both Guatemala and Tanzania, the two countries with DHS that include nutritional data for both children and adults. These changes seem reasonable in light of the 2016 obesity estimates from WHO, with the prevalence of obesity among children and adolescents (5–19 years old) being 9.9% in Guatemala and 2.5% in Tanzania, and 21.2% and 8.4%, respectively, among adults (18 years old and above) (WHO, 2020).

The prevalence of child mortality in the six middle-income countries is low in the global MPI, but adding health insurance appeared to be a considerably more demanding requirement. In Guatemala, Iraq and Tanzania—the countries with data on health insurance—the percentage of people living in a household with that deprivation climbed sharply to over 90%.^8^ While in Guatemala and Tanzania data were collected from both men and women, in the four other pilot countries, information about health insurance for male household members was not recorded and underreporting can thus be assumed. Basic healthcare provided by the government also varies across the pilot countries, and in Iraq the service is free, possibly disincentivizing households taking out private insurance, something which is reflected in the data.

Increasing of the number of years of schooling required from six to nine years and adding a gender criterion leads to substantial changes. Table 4 presents a comparison of the achievements in the six countries, showing household-level data for different schooling levels, one or two members, and with and without accounting for gender.^9^

**Table 4:** Cross-tabs of differences in educational achievements. *Source*: Authors’ calculations. Percentages refer to eligible reference population with observable data only

	Proportion of people (%) living in a household with at least:					
	One member with 6 years of schooling (global MPI)	One member with 9 years of schooling	Two members with 6 years of schooling	Two members with 9 years of schooling	One woman and one man with 6 years of schooling	One woman and one man with 9 years of schooling
Bangladesh	78.8	54.8	53.6	30.7	45.9	23.4
Guatemala	79.5	48.4	58.2	27.9	50.7	23.5
Iraq	88.7	60.9	72.1	38.4	61.7	26.7
Serbia	97.4	90.9	91.2	78.2	93.5	75.9
Tanzania	87.5	35.9	67.9	17.6	64.1	14.4
Thailand	88.1	75.1	74.7	50	72.1	47.7

Three observations demonstrate the added value of the new indicator. First, there is a large difference for most countries between six or nine years of schooling, either for one or any two household members, indicating that achieving universal lower secondary education is still a long way in many developing countries. Second, the numbers decline when the definition is changed from any *one* member to any *two* members, with the largest decrease in Bangladesh (25.2 p.p.) for six years and in Thailand (25.1 p.p.) for nine years of schooling.

The final observation relates to intrahousehold differences around having any two members or two members of different genders with six or nine years of schooling. The gender gap shows similar levels for both six and nine years, with a smaller decrease for most countries when moving to the higher level of achievement.^10^ In Iraq, however, we see a slight increase in the gap with the move to nine years, while in Guatemala the gender gap reduces as the level of required education increases. For the final MMPI indicator, we selected the gendered criteria with a minimum requirement of nine years (equivalent to lower secondary). Serbia is the only country where the majority of the population fulfil this criterion, reinforcing the need for continued global action to provide universal secondary education and minimize gender-based differences in educational achievement.

The new or increased MMPI deprivation thresholds also produced interesting findings for the living standards indicators. For instance, while less than 3% of Bangladesh’s population live in households that do not have access to improved sources of drinking water, the MMPI identifies 90% of people living in households that lack access to piped water on their premises, a requirement in the SDGs. Adding internet to complement access to electricity reveals a wide gap in access to information technology as many people are left offline, as illustrated in Guatemala (Fig. 1). For the housing indicator, there is a varied impact across countries based on the prevalence of overcrowding (see Table 10 in the Appendix). Many households who are non-deprived in the global MPI housing indicator are considered to be deprived in the MMPI due to the presence of overcrowding or a lack of finished housing materials. This increases the uncensored headcount ratios of the indicator across all countries, and particularly in Iraq and Thailand.^11^

### Poverty Results According to the MMPI

Table 5 presents the main results of the MMPI for the six pilot countries, accounting for the presence or absence of health insurance data in the child mortality indicator that affects cross-country comparability. Even across only six countries, there is significant variation in moderate poverty, with the MMPI ranging from 0.021 in Serbia to 0.692 in Tanzania. This confirms the intuition that lower middle-income countries, such as Tanzania, still have a long way to go to ensure sustainable poverty reduction across their populations, but even far more developed countries like Serbia have some gaps, made more visible by the new index, that need policy attention.

**Table 5:** MPI, incidence and intensity of poverty by MMPI. *Source*: Authors’ calculations

Country	MPI	Confidence Intervals (CI)		Headcount ratio (H)	CI		Intensity (A)	CI		Number of people in poverty, thousands (2018)
*Child mortality indicator includes data on health insurance*										
Guatemala	**0.569**	*0.560*	*0.579*	**87.6%**	*86.7%*	*88.5%*	**65.0%**	*64.4%*	*65.6%*	15,109
Iraq	**0.379**	*0.368*	*0.389*	**75.7%**	*74.1%*	*77.3%*	**50.0%**	*49.4%*	*50.6%*	29,109
Tanzania	**0.692**	*0.683*	*0.701*	**97.0%**	*96.4%*	*97.6%*	**71.4%**	*70.7%*	*72.1%*	54,630
*Child mortality indicator does not include data on health insurance*										
Bangladesh	**0.363**	*0.358*	*0.367*	**72.9%**	*72.2%*	*73.6%*	**49.7%**	*49.5%*	*49.9%*	117,652
Serbia	**0.021**	*0.017*	*0.025*	**5.3%**	*4.4%*	*6.2%*	**39.1%**	*37.9%*	*40.3%*	471
Thailand	**0.069**	*0.064*	*0.074*	**18.1%**	*17.0%*	*19.3%*	**38.1%**	*37.7%*	*38.5%*	12,585

Table 6 compares the key results with the most recent global MPI. As expected, both incidence and intensity increased in all countries, with large absolute increases for Tanzania, Guatemala, Iraq and Bangladesh, and smaller increases in Serbia and Thailand. Care must be taken in interpreting the moderate MPI findings due to the exclusion of health insurance in Bangladesh, Serbia and Thailand, and different definitions in Iraq. Nevertheless, the results suggest that the MMPI is likely to capture conditions (e.g. overcrowding, shared toilet, lack of bank account or internet) that are already being addressed in some countries but not in others, and highlight such differences between countries with similar levels of acute poverty in the global MPI.

**Table 6:** MPI, incidence and intensity of poverty by global MPI and MMPI. *Source*: Authors’ calculations. Global MPI estimates taken from Alkire et al. (2020a, 2020b)

Country	MPI		Headcount ratio (H)		Intensity (A)	
	Global MPI	MMPI	Global MPI (%)	MMPI (%)	Global MPI (%)	MMPI (%)
*Child mortality indicator includes data on health insurance*						
Guatemala	0.134	0.569	28.9	87.6	46.2	65.0
Iraq	0.033	0.379	8.6	75.7	37.9	50.0
Tanzania	0.273	0.692	55.5	97.0	49.3	71.4
*Child mortality indicator does not include data on health insurance*						
Bangladesh	0.104	0.363	24.6	72.9	42.2	49.7
Serbia	0.001	0.021	0.3	5.3	42.5	39.1
Thailand	0.003	0.069	0.8	18.1	39.1	38.1

**Fig. 5:**
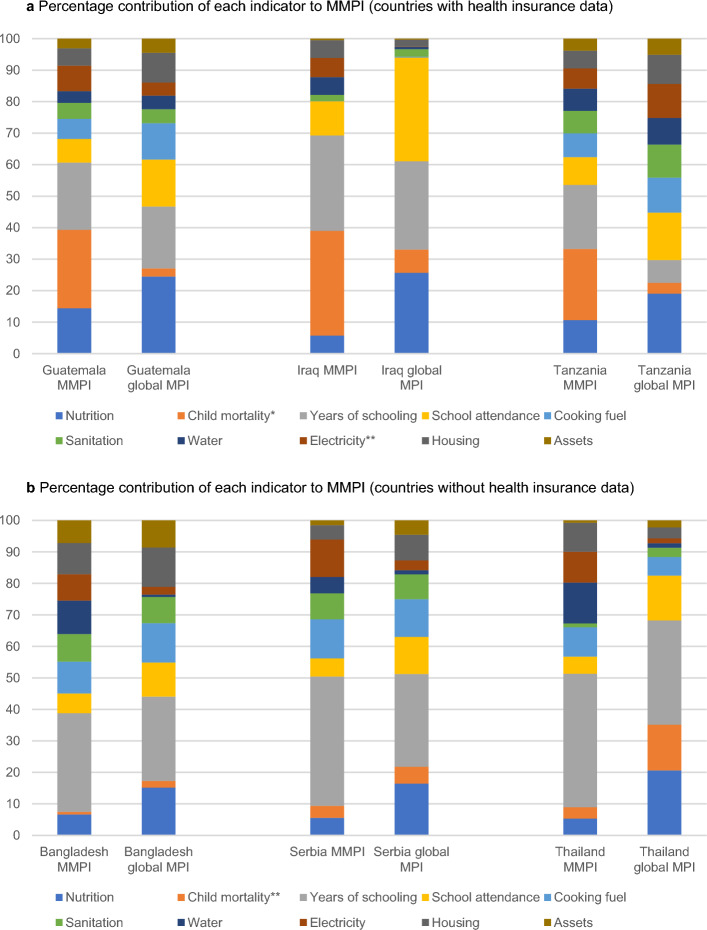
**a** Percentage contribution of each indicator to MMPI (countries with health insurance data). *Note*: The content of MMPI indicators match that of the global MPI (but apply higher cutoffs) with two exceptions. In the MMPI, the child mortality indicator also includes information on the presence of health insurance in the household (where data is available). In addition, the electricity indicator includes information on the internet access of the household. Source: Authors’ calculations. Global MPI estimates taken from Alkire et al. (2020a, 2020b). **b** Percentage contribution of each indicator to MMPI (countries without health insurance data). Note: The content of MMPI indicators match that of the global MPI (but apply higher cutoffs) with two exceptions. In the MMPI, the child mortality indicator also includes information on the presence of health insurance in the household (where data is available). In addition, the electricity indicator includes information on the internet access of the household. Source: Authors’ calculations. Global MPI estimates taken from Alkire et al. (2020a, 2020b)

Assessing the contribution of each indicator to poverty can provide important insights. Combined with additional data and expert analysis, it can help to design policies with the greatest impact on reducing moderate poverty. Figure 5a, b show the percentage contribution of indicators to the MMPI, with large variation across the six countries. In Thailand, deprivation in years of schooling is the main source of moderate poverty, contributing 42.4%. While most countries are on track to achieve the goal of universal primary education, when it comes to the employable skills and secondary education needed for meaningful engagement in society, education is still a serious issue.

Differences also appear with the global MPI, for instance in Tanzania, where the contribution of years of schooling to moderate poverty is substantially larger (20.3%) than the contribution to acute poverty (7.2%). In Guatemala, health-related indicators are more dominant, but it should be noted that the large contribution of child mortality to moderate poverty in Guatemala, Iraq and Tanzania reflects the large proportion of the population who lack health insurance, which is a requirement for this indicator in the MMPI. In Bangladesh, Serbia and Thailand, information on health insurance is not available, thus the contribution to overall poverty only reflects child mortality.

### Comparisons with Monetary Poverty

**Fig. 6:**
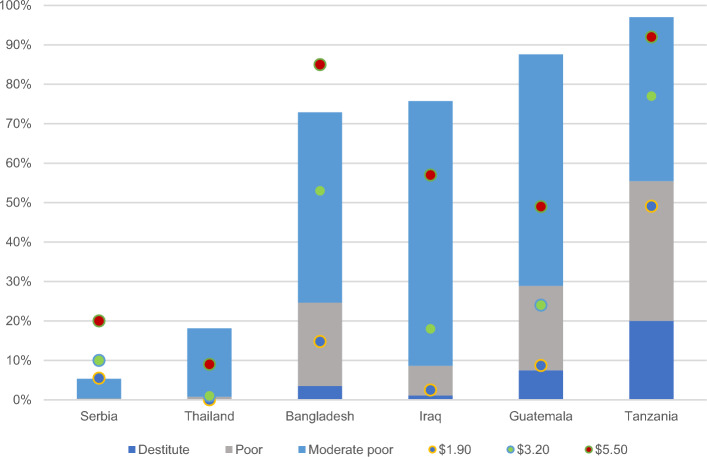
Incidence by different poverty measures (%). *Source*: Authors’ calculations. Global MPI estimates from Alkire et al. (2020a, 2020b). Monetary poverty estimates from the World Bank (n.d.). Latest available data for monetary figures are from 2018 (Thailand), 2017 (Serbia, Tanzania), 2016 (Bangladesh), 2014 (Guatemala), and 2012 (Iraq)

Figure 6 compares the incidence of poverty by the MMPI with existing international multidimensional and monetary poverty measures. The height of each bar represents the incidence of poverty using different versions of the MPI (destitute, acute, moderate), while the dots refer to the international monetary measures of poverty by the World Bank. Overall, the MMPI provides a meaningfully designed gradient of multidimensional poverty, ranging from a subset of acutely poor people in destitution, to the superset of acutely poor people in moderate poverty, displaying the differences in poverty levels due to changes in the deprivation cutoffs. While not strictly parallel, the three MPIs can be compared to the monetary measures to offer complementary information on the nature and level of poverty in a country.

As previous studies shown, multidimensional poverty measures and monetary measures capture different aspects of poverty and thus do not always result in similar poverty headcount ratios (Alkire et al., 2020b; Evans et al., 2020). Due to the small number of countries included and the aforementioned data concerns, no definitive pattern emerges about the relationship between monetary and multidimensional poverty. In four countries (Thailand, Iraq, Guatemala and Tanzania), incidence of the MMPI is higher than the $5.50 measure, with the reverse true in the other two countries (Serbia and Bangladesh). While the MMPI likely underestimates the level of poverty in Bangladesh due to the lack of health insurance in the child mortality indicator, the result for Serbia poses a more interesting finding that requires further investigation. In fact, all monetary measures—even the $1.90 a day—have a higher headcount ratio than the MMPI. Meanwhile, in Tanzania, more people are living in poverty according to the MMPI than according to any of the monetary measures.

These interesting empirical overlaps and diversions between the gradients point to the varying nature of poverty across country and development contexts, highlighting the added value of the an MMPI, but further analysis across a larger set of countries would be required to determine the relationship between monetary and multidimensional poverty using the MMPI.

### Disaggregation of Results

A key advantage of any MPI is the option to disaggregate results by population subgroups (as data allows) to provide a refined analysis of poverty across the population. The MMPI, like the global MPI, can be disaggregated by subnational regions, urban and rural areas, and age groups to reveal disparities in multidimensional poverty. More, the moderate MPI can provide visibility to the urban poor who are often not captured by acute measures such as the global MPI, especially in countries across Europe, Central Asia, East Asia and the Pacific, or Latin America and the Caribbean, where acute poverty in urban areas has been largely eliminated as a result of development.

Table 7 shows results by type of area, with a consistently higher incidence across rural areas for both moderate and acute poverty (UNDP & OPHI, 2020). However, the gap between rural and urban areas is less pronounced for moderate poverty. In the case of the countries with the lowest acute poverty levels (Serbia and Thailand), the MMPI seems to offer a meaningful increment and identifies a new layer of ‘moderately poor’ across both urban and rural areas, using the cutoffs for higher levels of achievements.

**Table 7:** Disaggregation of MMPI, incidence and intensity by urban and rural areas. *Source*: Authors’ calculations. Global MPI estimates from Alkire et al. (2020a, 2020b)

Country	Area	Moderate MPI			Global MPI		
		MPI	H (%)	A (%)	MPI	H (%)	A (%)
*Child mortality indicator includes data on health insurance*							
Guatemala	Rural	0.669	96.0	69.6	0.193	41.3	46.6
Guatemala	Urban	0.434	76.1	57.0	0.053	11.9	44.3
Iraq	Rural	0.468	87.3	53.6	0.05	12.6	39.3
Iraq	Urban	0.339	70.6	48.0	0.025	6.9	36.7
Tanzania	Rural	0.744	99.1	75.1	0.34	67.6	50.3
Tanzania	Urban	0.564	91.8	61.4	0.112	26.0	43.2
*Child mortality indicator includes data on health insurance*							
Bangladesh	Rural	0.397	79.2	50.2	0.233	48.6	47.9
Bangladesh	Urban	0.236	50.1	47.2	0.103	23.0	44.9
Serbia	Rural	0.036	9.4	38.0	0.003	0.7	42.8
Serbia	Urban	0.011	2.5	42.0	0.001	0.1	41.4
Thailand	Rural	0.089	23.3	38.1	0.004	1.0	38.9
Thailand	Urban	0.046	12.1	38.0	0.002	0.5	39.5

**Fig. 7:**
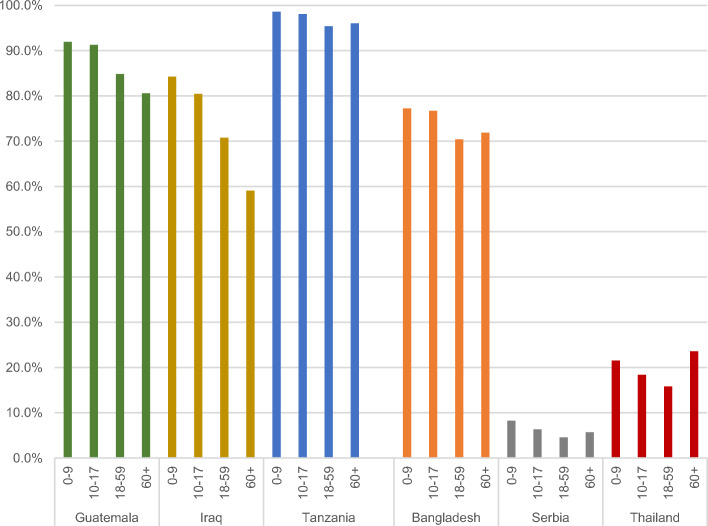
Poverty incidence by age cohort (%). *Source*: Authors’ calculations

Looking at age groups (Fig. 7), children under 18 tend to be the most affected by acute poverty across the world (UNDP & OPHI, 2020). This is maintained in the MMPI, with children having the highest levels of moderate poverty in every country except Thailand. However, the differences between the age groups are less pronounced than in the global MPI. The greatest difference is in Iraq, where 84.3% of children aged 0–9 and 59.1% of adults aged 60 and over are poor.

**Fig. 8:**
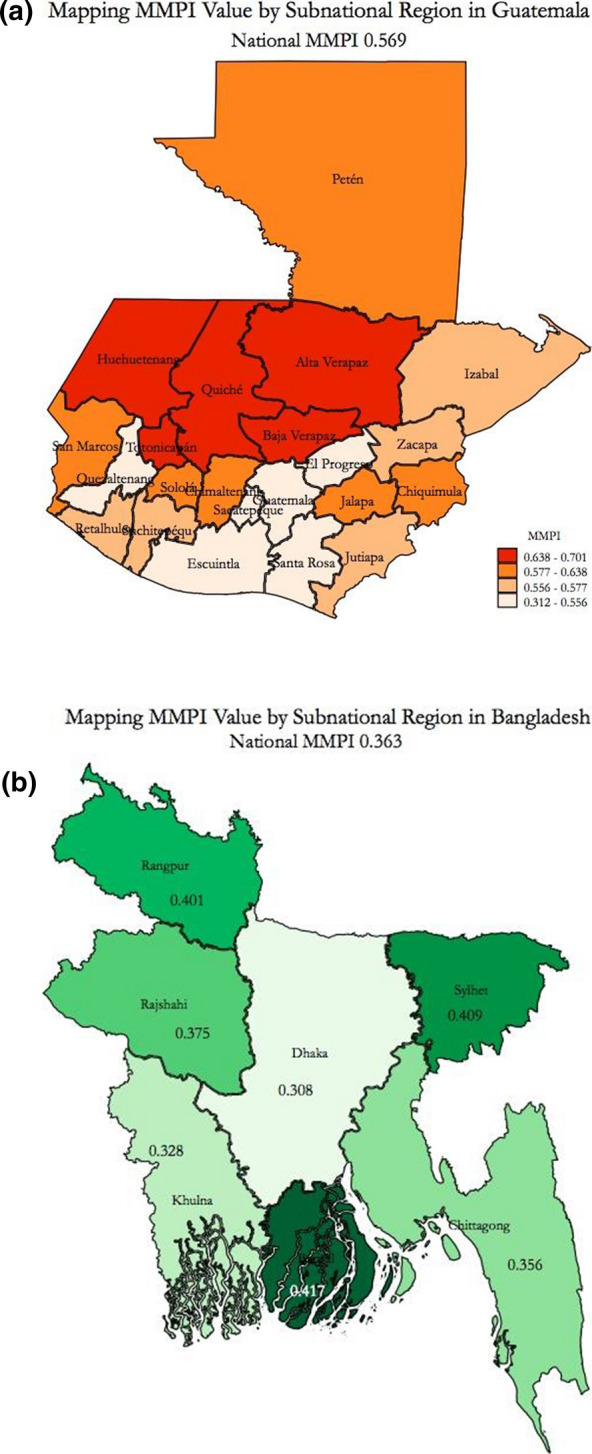
**a**, **b** MMPI by subnational region in Guatemala and Bangladesh. *Source*: Authors’ compilation, using underlying shapefiles from DIVA-GIS (www.diva-gis.org/gdata). *Note*: The Bangladesh region of Mymenshingh is not depicted on the map (it has an MMPI of 0.442)

The MMPI, like the global MPI, is also disaggregated by subnational regions (see Table 11 in the Appendix) to reveal poverty variations within a country, enabling more focused and tailored analysis and responses. Differences across regions vary by country, with poorer countries showing higher levels of regional disparity, suggesting that there is a further benefit of disaggregating the MMPI at the subnational level. In Serbia, the country with the lowest level of moderate poverty (0.021), the MMPI ranges from 0.006 in Belgrade to 0.030 in South and East Serbia. Figure 8 presents subnational results in Bangladesh, the third-poorest country (0.363), and Guatemala, the poorest middle-income country (0.569). Both show variation in their MMPI at the subnational level, but while a clear pattern emerges in Guatemala, with moderate poverty more prevalent in the north and north-western regions (Fig. 8a), poverty in Bangladesh appears to be less geographically concentrated (Fig. 8b).

## Conclusions and Way Forward

Our proposed Moderate Multidimensional Poverty Index (MMPI) reflects the spirit of Agenda 2030, and in particular the aspiration to eradicate poverty in all its forms, everywhere. While the need for a moderate multidimensional poverty measure has been widely recognized, such a measure has not yet been broadly applied. The MMPI increases the deprivation cutoff for 9 of the 10 indicators of the global MPI with the intention of initiating a discussion on estimating this or a similar measure globally.

The new MMPI has significant policy relevance. At the global level, it monitors efforts towards the increased level of ambition in the SDG agenda. At the national level, the MMPI allows international benchmarking and sheds light on the remaining deprivations that stand in the way of sustainable poverty reduction. Moreover, like all MPIs, the MMPI provides a tool for policy makers to plan required policy actions, using subgroup decomposition to highlight the poorest groups and guide efforts towards deprivations that most effectively reduce poverty.

Data limitations still inhibit our ability to fully measure and track progress towards every person’s right to lead a life free from poverty. Important dimensions of poverty such as human security, employment, environment, and shocks, are not included in the internationally comparable surveys and thus cannot be included in this MMPI. Differences in coverage and measurement of indicators such as nutrition, health care, and internet access in DHS and MICS, also present a challenge for comparability. Newer surveys are likely to close this data gap for health insurance, but further efforts are needed to improve data availability.

To further advance Agenda 2030, we suggest that a new moderate index for multidimensional poverty is added to the human development indicator family. In proposing the structure of the new MMPI presented here, we recognize that the indicators will require ongoing improvement. While the MMPI will not replace the global MPI, it sets a realistic level of ambition beyond the more acute measures and creates a superset that adds to existing knowledge on multidimensional poverty. The MMPI also does not replace other efforts to expand the scope of metrics with new dimensions, or to generate regional multidimensional indices. Instead, the MMPI presents an index that is globally comparable, anchored in the common goals articulated in Agenda 2030, and allows for the inclusion of intrahousehold deprivations and decomposition of results by subgroups.

This paper shows that constructing a global MMPI is possible, and demonstrates its value-added by showing new aspects of poverty—such as gendered disparities in education, and intraregional variation—across countries, that are not captured by existing measures. Empirical trials with the emerging newer datasets are needed to ensure that all indicators are robust and reliable before the MMPI can be finalized and adopted for wider use.

## Data Availability

Not applicable.

## Appendix

See Tables

**Table 8:** Data availability for revised indicators. *Source*: Authors’ calculations

Country	Survey	Year	Nutrition	Child mortality	Years of schooling	School attendance	Cooking fuel	Sanitation	Drinking water	Electricity
Bangladesh	MICS	2019	Children under 5 years	No male questionnaire; no data on health insurance						
Guatemala	DHS	2014/15	Children under 5 year and women aged 15–49	Health insurance data for women aged 15–49 and a half subset of men aged 15–54 (those who answered the men's questionnaire)						
Iraq	MICS	2018	Children under 5 years	No male questionnaire; all data on child mortality and health insurance are from women						
Serbia	MICS	2014	Children under 5 years	No birth history file or male questionnaire; child mortality is from all years, not past 5 years, and only as reported by women; no data on health insurance						
Tanzania	DHS	2015/16	Children under 5 years and women aged 15–49							No data on internet, so only captures electricity
Thailand	MICS	2015/16	Children under 5 years	No data on health insurance						No data on internet, so smartphone used instead

8,

**Table 9:** Percentage of the population that is deprived in a given indicator (regardless of their poverty status) for global MPI and MMPI. *Source*: Authors’ calculations. Global MPI estimates taken from Alkire et al., (2020a, 2020b)

Country	Nutrition	Child mortality	Years of schooling	School attendance	Cooking fuel	Sanitation	Water	Electricity	Housing	Assets
*Child mortality indicator includes data on health insurance*										
**MMPI**										
Guatemala	50.3	92.7	73.8	25.5	67.0	52.8	46.2	89.8	57.5	31.3
Iraq	13.1	97.7	69.9	24.6	0.4	14.5	46.9	46.1	43.7	3.3
Tanzania	44.3	96.3	84.5	36.5	96.5	90.1	89.5	80.4	70.0	47.4
*Child mortality indicator does not includes data on health insurance*										
Bangladesh	15.6	1.9	74.0	14.0	81.4	64.3	89.9	60.8	74.0	49.6
Serbia	1.6	1.5	18.5	1.0	34.4	8.0	17.8	32.6	5.3	0.7
Thailand	4.2	2.1	52.4	3.0	21.5	2.9	72.1	24.7	28.3	1.2
**Global MPI**										
Bangladesh	15.5	1.9	21.2	9.0	81.4	35.7	2.9	7.8	70.3	29.5
Guatemala	31.2	2.8	20.5	16.4	67.0	21.4	40.1	13.0	41.2	14.0
Iraq	12.7	3.1	11.3	17.2	0.4	8.2	1.9	0.1	7.9	0.4
Serbia	1.3	1.5	2.6	0.5	34.3	3.1	2.1	0.3	2.4	0.3
Tanzania	35.8%	6.4%	12.5%	27.4%	96.5%	79.9%	59.3%	80.3%	62.7%	31.0%
Thailand	3.7%	2.1%	11.9%	1.1%	21.5%	2.8%	1.9%	0.3%	3.6%	0.6%

The content of MMPI indicators match that of the global MPI (but apply higher cutoffs) with two exceptions. In the MMPI, the child mortality indicator also includes information on the presence of health insurance in the household (where data is available). In addition, the electricity indicator includes information on the internet access of the household

9,

**Table 10:** The impact of overcrowding on the housing indicator. *Source*: Authors’ calculations

	Households deprived in overcrowding (Frequency)	Households deprived in overcrowding (%)	Households deprived in overcrowding but non-deprived in housing (global MPI) (Frequency)	Households deprived in overcrowding but non-deprived in housing (global MPI) (%)	Uncensored headcount ratio housing (global MPI) (%)	Uncensored headcount ratio housing (MMPI) (%)
Bangladesh	42,810	17.3	6653	15.5	70.26	74.0
Tanzania	16,635	27.4	4346	26.1	62.71	70.0
Iraq	52,936	40.7	47,253	89.3	7.93	43.7
Thailand	17,207	16.6	15,550	90.4	3.63	28.3
Guatemala	40,863	41.7	15,165	37.1	41.21	57.5
Serbia	1248	6.1	1,132	90.7	2.36	5.3

^10^,

**Table 11:** MMPI results by subnational regions in six countries: MMPI, H, A and percentage contributions. *Source*: Authors’ calculations

Country	Subnational region	MMPI	H (%)	A (%)	Nutrition (%)	Child mortality (%)	Years of schooling (%)	School attendance (%)	Cooking fuel (%)	Sanitation (%)	Water (%)	Electricity (%)	Housing (%)	Assets (%)
*Child mortality indicator includes data on health insurance*														
Tanzania	Central	0.723	99.1	73.0	9.3	21.3	20.7	8.1	7.6	7.4	7.3	7.2	6.3	4.8
Tanzania	Eastern	0.581	92.0	63.1	11.7	25.7	20.3	7.7	8.0	6.7	7.8	4.8	4.3	3.0
Tanzania	Lake	0.742	98.8	75.1	10.8	21.8	20.0	9.8	7.4	7.1	7.1	6.6	5.9	3.5
Tanzania	Northern	0.631	94.8	66.6	11.8	23.8	21.2	6.3	7.8	7.5	6.2	6.0	5.3	4.1
Tanzania	South West Highlands	0.724	98.9	73.2	10.5	22.1	20.4	8.8	7.5	7.3	7.1	6.8	5.1	4.6
Tanzania	Southern	0.711	98.6	72.2	8.2	22.6	21.1	8.3	7.6	7.3	7.4	7.0	6.5	3.9
Tanzania	Southern Highlands	0.659	97.1	67.8	9.9	23.8	21.3	6.2	8.1	7.3	7.2	6.8	5.1	4.2
Tanzania	Western	0.767	99.0	77.5	10.3	20.5	20.1	12.0	7.2	6.9	6.8	6.7	6.2	3.3
Tanzania	Zanzibar	0.542	88.9	61.0	14.6	27.3	15.3	7.2	8.8	7.1	6.0	5.2	4.9	3.5
Iraq	Anbar	0.392	81.5	48.1	5.1	34.6	32.0	10.4	0.0	0.6	0.9	8.3	6.8	1.2
Iraq	Babil	0.407	75.6	53.8	4.2	30.9	28.4	12.0	0.0	3.9	7.3	7.4	5.5	0.4
Iraq	Baghdad	0.353	71.0	49.7	7.7	33.4	29.5	11.9	0.0	1.3	6.1	4.9	4.8	0.3
Iraq	Basrah	0.433	80.4	53.9	6.5	30.8	27.4	12.5	0.0	3.0	10.2	4.0	5.2	0.3
Iraq	Diala	0.373	77.5	48.1	3.4	34.6	32.4	9.3	0.1	0.9	5.6	7.4	5.9	0.4
Iraq	Duhok	0.301	68.8	43.8	5.5	38.0	34.6	9.6	0.1	0.4	0.6	3.8	7.2	0.3
Iraq	Erbil	0.295	68.5	43.1	3.3	38.7	36.7	8.6	0.0	0.7	0.8	4.1	7.0	0.1
Iraq	Karbalah	0.410	78.3	52.4	5.2	31.6	29.7	10.3	0.0	2.2	8.9	6.0	5.8	0.3
Iraq	Kirkuk	0.284	62.1	45.8	5.5	36.1	34.2	9.9	0.0	1.6	1.5	6.9	3.6	0.7
Iraq	Misan	0.482	88.5	54.5	6.3	30.3	27.3	12.5	0.0	3.0	8.2	6.0	5.8	0.5
Iraq	Muthana	0.484	90.9	53.3	6.1	31.0	26.8	11.6	0.0	1.5	8.9	6.7	6.9	0.4
Iraq	Nainawa	0.375	76.2	49.2	6.5	33.9	31.8	11.5	0.0	1.2	0.6	7.4	5.9	1.2
Iraq	Najaf	0.429	81.2	52.8	5.5	31.4	28.5	11.5	0.6	3.5	8.0	6.0	4.9	0.2
Iraq	Qadisyah	0.416	79.5	52.3	6.1	31.7	27.6	11.5	0.1	4.6	6.6	6.8	4.5	0.5
Iraq	Salahaddin	0.366	77.9	47.0	4.2	35.3	32.9	10.8	0.0	1.0	2.2	8.4	4.8	0.5
Iraq	Sulaimaniya	0.267	63.2	42.3	2.8	39.4	37.8	5.4	0.0	0.7	2.3	5.6	5.8	0.2
Iraq	Thiqar	0.440	81.5	54.0	7.9	30.8	26.7	9.4	0.0	3.3	8.9	7.3	5.6	0.2
Iraq	Wasit	0.438	81.2	53.9	4.2	30.7	28.5	11.2	0.1	2.8	8.4	7.2	6.4	0.5
Guatemala	Guatemala Municipio	0.312	61.7	50.6	19.2	30.9	21.4	5.0	0.7	2.7	7.1	8.3	4.0	0.8
Guatemala	Guatemala Resto	0.419	76.0	55.1	16.5	28.5	22.7	5.1	3.1	3.6	5.5	8.8	4.8	1.4
Guatemala	El Progreso	0.511	86.0	59.4	14.3	27.4	23.1	4.8	6.4	4.5	3.1	8.8	5.0	2.7
Guatemala	Sacatepéquez	0.473	80.5	58.7	16.0	27.5	22.5	6.2	5.3	3.2	3.4	8.7	5.1	2.1
Guatemala	Chimaltenango	0.578	89.5	64.5	15.4	25.2	21.6	8.2	6.8	5.0	2.7	8.2	4.6	2.2
Guatemala	Escuintla	0.522	86.5	60.3	14.4	24.8	23.8	5.9	5.6	4.2	5.1	8.8	5.4	1.9
Guatemala	Santa Rosa	0.553	88.4	62.5	12.4	26.4	22.6	6.4	6.8	4.3	3.8	8.7	5.7	3.0
Guatemala	Sololá	0.605	94.0	64.4	14.5	25.5	20.4	7.5	8.1	5.9	1.4	8.3	4.6	3.6
Guatemala	Totonicapán	0.639	94.8	67.4	14.6	24.5	20.3	8.3	7.6	6.4	2.5	8.0	5.5	2.3
Guatemala	Quetzaltenango	0.556	87.3	63.7	14.4	25.8	21.2	8.2	6.9	5.0	3.2	8.3	4.8	2.1
Guatemala	Suchitepéquez	0.577	90.4	63.8	14.0	24.1	22.2	6.7	6.6	4.4	5.3	8.3	5.8	2.6
Guatemala	Retalhuleu	0.575	90.5	63.6	13.6	25.0	20.6	6.1	7.3	5.5	5.9	8.4	5.7	1.9
Guatemala	San Marcos	0.638	93.8	68.0	13.8	24.3	21.1	8.7	7.4	5.2	2.8	7.9	5.6	3.2
Guatemala	Huehuetenango	0.689	94.0	73.3	15.1	22.4	20.7	9.8	7.0	5.6	2.4	7.4	5.5	4.1
Guatemala	Quiché	0.685	95.3	71.9	14.9	23.0	20.6	8.6	7.3	6.0	2.0	7.5	6.3	3.8
Guatemala	Baja Verapaz	0.639	94.4	67.7	13.0	24.3	21.4	7.7	7.1	5.8	3.1	7.9	6.0	3.8
Guatemala	Alta Verapaz	0.701	95.5	73.4	12.6	22.5	20.1	8.3	7.2	6.0	4.4	7.4	6.5	5.0
Guatemala	Petén	0.636	93.8	67.8	12.8	24.2	19.9	6.9	7.2	6.4	5.3	7.9	6.1	3.4
Guatemala	Izabal	0.558	87.4	63.8	12.9	25.3	21.6	7.6	6.2	5.1	3.7	8.2	6.2	3.2
Guatemala	Zacapa	0.566	88.5	64.0	13.6	25.6	22.5	6.9	6.5	4.5	3.3	8.3	5.6	3.3
Guatemala	Chiquimula	0.629	91.0	69.2	13.9	23.9	21.7	7.8	6.7	4.7	3.3	7.7	6.0	4.3
Guatemala	Jalapa	0.627	91.5	68.5	13.4	24.1	21.6	8.3	6.9	5.2	3.0	7.9	5.9	3.7
Guatemala	Jutiapa	0.575	91.1	63.2	13.3	25.7	22.3	5.7	7.3	4.9	3.9	8.4	5.6	3.0
*Child mortality indicator includes data on health insurance*														
Serbia	Belgrade	0.006	1.6	37.4	9.8	0.7	41.6	18.6	8.0	3.3	5.5	7.5	4.0	0.9
Serbia	South And East Serbia	0.030	8.1	37.8	3.3	3.1	42.2	1.4	14.5	10.8	5.2	13.8	4.1	1.8
Serbia	Sumadija And West Serbia	0.021	5.8	36.8	2.7	3.1	43.9	4.8	14.2	8.1	6.3	12.9	2.8	1.1
Serbia	Vojvodina	0.024	5.5	43.7	9.8	5.9	37.1	8.8	9.4	6.7	4.0	9.9	6.8	1.6
Thailand	Bangkok	0.013	3.5	37.1	3.8	3.3	43.1	11.9	0.6	7.5	9.3	7.9	9.9	2.6
Thailand	Central	0.044	11.3	38.9	5.5	3.9	41.8	8.7	4.6	2.0	13.2	9.7	9.6	1.0
Thailand	North	0.109	28.6	38.0	3.5	4.4	42.8	4.6	10.9	1.0	11.4	9.9	10.7	0.7
Thailand	Northeast	0.110	29.2	37.5	4.9	2.7	42.7	3.4	12.7	0.4	14.3	10.2	8.2	0.4
Thailand	South	0.048	12.3	39.3	12.6	5.0	40.5	8.4	1.7	1.7	11.9	8.4	8.6	1.1
Bangladesh	Barishal	0.417	81.2	51.3	6.6	0.7	29.2	4.6	10.7	10.0	10.8	8.7	10.3	8.6
Bangladesh	Chattogram	0.356	70.5	50.5	7.8	1.1	30.4	7.8	9.7	8.6	10.5	7.3	9.6	7.3
Bangladesh	Dhaka	0.308	64.5	47.8	6.7	0.7	33.2	7.2	8.6	9.1	10.0	8.0	10.2	6.4
Bangladesh	Khulna	0.328	70.2	46.7	5.2	0.7	33.6	4.2	11.7	8.2	11.8	8.8	9.8	6.2
Bangladesh	Mymenshing	0.442	83.4	53.0	6.7	0.6	29.4	7.9	10.2	8.6	10.3	8.6	10.0	7.7
Bangladesh	Rajshahi	0.375	76.9	48.8	5.6	0.6	32.2	4.3	11.1	8.9	10.9	9.3	10.1	7.0
Bangladesh	Rangpur	0.401	80.4	49.8	5.6	0.7	30.8	4.1	11.0	8.8	11.0	9.9	10.4	7.7
Bangladesh	Sylhet	0.409	75.5	54.1	9.1	1.5	29.0	9.1	9.8	7.7	10.0	7.0	8.9	8.0

The content of MMPI indicators match that of the global MPI (but apply higher cutoffs) with two exceptions. In the MMPI, the child mortality indicator also includes information on the presence of health insurance in the household (where data is available). In addition, the electricity indicator includes information on the internet access of the household

11

## Footnotes

1 Measured using constant international US dollars in purchasing power parity (PPP) terms, with 2017 as the base year.

2 A person is considered poor if they live on less than US$1.15 plus half of the value of median consumption (or income) per day in their country.

3 Estimates based on World Bank (2022).

4 In fact, many European countries submitted their comparable AROPE measure against SDG 1.2.2, and two countries submitted the global MPI as their national measure.

5 If one gender is absent, the household is assessed based on two members of the same gender; if it is a singleton household, the singleton must have completed the required years of schooling.

6 Technically it would be possible for a household to only own a washing machine and a bank account, violating the superset logic by being (non-)deprived according to the MMPI and the global MPI. However, empirically we did not observe this to be the case in the six pilot countries.

7 Note that the Venn diagrams on health insurance is presented in two forms as the indicator carries over 60% missing values. The first Venn shows the percentages of the eligible reference population on observable data only. The second Venn presents missing values on health insurance in line with the moderate MPI, where missing values on “assets” – and insurance is seen as an intangible asset—are interpreted – in line with the global MPI—as a non-attainment (for reference, see Alkire & Santos, 2014 and Vollmer & Alkire, 2022). Put differently, it is assumed that a missing value on insurance means not having insurance. This is done to present a lower bound measure of poverty, in order to avoid overestimation of the non-deprived populations. Both options on the Venn diagram are presented for consistency, accuracy, and transparency.

8 Including the interpretation of missing values as a non-attainment in the MMPI, in alignment with the treatment of missing values in the global MPI.

9 As with the Venn diagrams, the percentages presented here refer to the eligible reference population with observable data only. For example, in the column on the achievements of households with two educated members, the percentages only refer to households with two or more eligible members. Note that this is different from the coding of the final indicator that considers additional conditions (as described in Table 2).

10 Note that the observed increase in the achievement of households with one educated female and one educated male member in Serbia, compared to any two educated members with six years of schooling, is due to household composition. Notably, a high percentage (86.2%) of women in the reference population have six years of schooling. However, the percentage of women with nine years of schooling is lower (57.5%), hence we see a lower level of achievement in the gendered version, compared to any two members with nine years of schooling.

11 Note that the revised MMPI housing indicator also uses an updated floor definition, whereby a ‘household is considered deprived if it has a floor that is natural or rudimentary material’. For Thailand, this results in a large increase of the uncensored headcount ratio as wood planks are a common floor material (17.6% of households reported wood planks as their floor material).
